# A Case of Aneurism of the Heart, Pericarditis, and Peritonitis Hepatica, Followed by Ascites, Which, after a Violent Attack of Sciatica, Terminated in Paralysis of the Abdominal Viscera and Death

**Published:** 1814-12

**Authors:** L. B. Guersent


*For the Medical and Physical Journal.*

### *A Case of Aneurism of the Heart, Pericarditis, and Peritonitis Hepatica, followed by Ascites, which, after a violent Attack of Sciatica, terminated in Paralysis of the Abdominal Viscera and Death;* by L. B. Guersent, D. M. P.

THIS disease commenced about the thirtieth year of the patient's age, with symptoms of organic affection of the heart—her respiration was short and interrupted, and at times her legs swelled. At the age of thirty-eight, two months after a favourable accouchment, she was attacked with fever, oppression, pain in the mediastinum, and puffing of the belly and legs, which were reduced by leeches, blistering the thighs, and solution of the acetate of potash, &c. The symptoms, however, returned a month afterwards with greater violence, which were calmed by bleeding in the foot; but the swelling of the legs returned, and shortly afterwards ascites made its appearance, which for three years continued to increase, although squills, digitalis, &c. were freely prescribed, without disturbing the animal functions. Her digestion was in general good; the alvine evacuations abundant, watery, and bilious. She menstruated regularly, and there never was any fever, but always had considerable palpitation of the heart, and which was distinctly visible in two carotids: the jugular veins were very large, with great determination of blood to the head, particularly about the menstrual periods. At the commencement of January 1813, she had been tapped twenty-eight times, at which time, she lost her appetite, could not sleep, was troubled with repeated fits of coughing, and considerable oppression. The abdomen being very large, she was tapped on the 10th of January, being the twenty-ninth time. The cough immediately subsided, but in the night she was attacked with violent pain in the loins, which extended to the thighs. On the 12th and 13th the pain was so great, that large doses of opium, frictions of camphor and opium, afforded no relief, and paralysis of the inferior extremities of the bladder and rectum supervened. In spite of sinapisms, blisters, irritating frictions, and the most powerful tonics, she died on the 14th, with all her moral faculties perfect.

On dissection, the heart was found enlarged to double its ordinary size, all the cavities very much dilated, and the parietes extremely thin; the auriculo-ventricular orifice was greatly diminished from ossification of the sigmoïd valves; the pericardium adhered completely to the heart; the abdomen contained about two French quarts of a yellow muddy serum; the liver was but little enlarged, and entirely covered with a very thick membraniform albuminous coat.

